# Elucidating CYP2D6-driven metabolism and hepatotoxic bioactivation of metoprolol in plateable human and animal hepatocytes

**DOI:** 10.5599/admet.2961

**Published:** 2025-10-30

**Authors:** Jiang Pu, Mei Yang, Min Zhang, Ruiqi Gao, Yue Xiao, Lingyu Liu, Chuanjing Zhang, Wennuo Xu, Kaifang Li, Wanyong Feng

**Affiliations:** Bioduro Biologics Co., Ltd., Shanghai, China

**Keywords:** Metabolite, cytotoxicity, recombinant, low-clearance, glutathione, CYP2D6

## Abstract

**Background and purpose:**

As a classic β-blocker with low systemic clearance, metoprolol has been linked to rare but clinically significant hepatotoxicity, yet its hepatic metabolic fate remains poorly characterized.

**Experimental approach:**

Metoprolol was incubated individually in plateable human and animal hepatocytes, and recombinant cytochrome *(*CYP) P450 enzymes, followed by sample processing for cytotoxicity assessment, stability analysis, phenotyping and metabolite identification studies.

**Key results:**

*In vitro* cytotoxicity assessment revealed distinct species-specific responses to metoprolol exposure. Metoprolol showed no observable cytotoxicity across the tested concentration range (0 to 500 μM) in human hepatocytes, whereas it was cytotoxic only at a concentration of 500 μM in rat hepatocytes. Metabolic characterization showed low intrinsic clearance in human hepatocytes (0.56±0.12 μL min^-1^ *per* million cells) over a 72-hour incubation. Comprehensive mass spectrometer analysis identified 22 metabolites across four species (rat, dog, monkey, and human) and fifteen metabolites were identified as the new ones, with CYP2D6-mediated biotransformation pathways (including mono-oxygenation, *O*-demethylation, and oxidation) accounting for the generation of four major metabolites (M1, M10, M13, M17). Notably, species-specific metabolism was observed for s-hydroxy-metoprolol (M10). It served as the predominant metabolite in rat hepatocytes and underwent subsequent bioactivation to a reactive glutathione (GSH) conjugate. Inhibition studies with 1-aminobenzotriazole (a non-specific CYP inhibitor) confirmed the CYP-dependent nature of this hepatotoxic metabolic pathway.

**Conclusion:**

The sustained metabolic activity of plateable hepatocytes facilitated a comprehensive metabolic profiling of metoprolol, including direct observation of GSH-mediated bioactivation. Integrating with cytotoxicity data, these findings offered crucial insights into its hepatic adverse effects.

## Introduction

Hepatic metabolism is the primary pathway for clearing xenobiotic compounds from the body. Approximately 70 % of marketed pharmaceuticals are metabolized in the liver through its detoxification functions [1,2]. It is well established that high hepatic clearance often results in low systemic bioavailability. Conversely, a detailed understanding of hepatic metabolism can support drug optimization efforts by revealing metabolic soft spots in lead compounds [3]. Consequently, hepatocytes play an indispensable role in drug discovery.

Hepatocytes are the preferred *in vitro* model for metabolism studies due to their complete complement of phase I and II enzymes, which accurately recapitulate intrinsic clearance mechanisms [4-13]. These cells also express the full spectrum of hepatic transporters that govern drug uptake and efflux, providing a physiologically relevant platform for disposition studies [14,15]. Notably, comparative metabolite profiling across species-specific hepatocytes enables prediction of interspecies metabolic differences, thereby informing appropriate animal model selection for preclinical toxicology assessments [16,17].

Recent years have witnessed notable advancements in hepatocyte systems for low-clearance compound assessment, including suspended hepatocytes and adherent primary hepatocytes, HepaRG cells [18-25], and various hepatocyte-stromal cell cocultures [26-47]. Adherent primary hepatocytes are optimal for low-clearance compound studies due to their stable metabolic function and intact enzyme systems in extended cultures. Traditional hepatocellular carcinoma cell lines are limited by genetic instability and progressive phenotypic drift, and coculture systems present operational challenges due to the biological complexity imposed by heterologous cellular components. Although suspended hepatocytes are commonly used for metabolic screening, their utility is severely hampered by the rapid loss of enzymatic activity within 4 hours [25,42,48]. Methods like the relay technique [24] extend observation periods via sequential incubations but complicate routine application [42,49]. In contrast, adherent primary hepatocytes maintain robust metabolic enzyme activity and intact hepatic enzymatic profiles without artificial modification, rendering them the most physiologically relevant *in vitro* model for prolonged clearance studies [50-52]. Compelling evidence from Griffin *et al.* [53] demonstrated superior performance of plateable rat hepatocytes compared to suspended cells for low-clearance measurements. Subsequent human hepatocyte studies have validated the reliability of adherent systems for evaluating low-clearance compounds [20,54].

Despite being extensively utilized as both a CYP2D6 phenotyping probe and a model high-permeability compound exhibiting excellent oral bioavailability [55-59], metoprolol's complete metabolic disposition as a low-clearance agent [60,61] remains insufficiently characterized, particularly regarding its hepatic elimination pathways. Existing metabolite identification studies are conducted in various matrices, including rat plasma [62,63], human liver microsomes, plasma, and urine [57,64-68], as well as dog plasma [69]. Only minor metabolites are reported *in vivo*, a fact attributed to the significant renal excretion. Moreover, even fewer metabolites are typically observed *in vitro* under short-term incubation conditions. This study, therefore, aims to systematically characterize metoprolol metabolism using plateable hepatocytes with longer extended culture and recombinant enzymes, providing novel insights into its hepatic adverse effects.

## Experimental

### Chemicals and reagents

Metoprolol (CAS: 51384-51-1, Lot: 6-JKL-52-4) was purchased from TRC Canada, while α-hydroxy-metoprolol (CAS: 56392-16-6, Lot: H2429287) and metoprolol acid (CAS: 56392-14-4, Lot: L2213483) were obtained from Aladdin. Hepatocytes from male Sprague-Dawley rats (HEP134054, 10 donors), male Beagle dogs (CDH100BE-V11201, 1 donor), male Cynomolgus monkeys (23D008, 1 donor), and male Caucasians (005, 1 donor) were sourced from Biopredict, TPCS, IPHASE, and XENOTECH, respectively. The kit of CellTiter-Glo® Luminescent Cell Viability Assay (Lot#: 0000582333) was purchased from Promega. Recombinant enzymes, including CYP1A2 (Lot#: C1A2R015A), CYP2D6 (Lot#: C2D6R040), CYP2C9 (Lot#: C2C9BR014), CYP2C19 (Lot#: C2C19BR027), CYP3A4 (Lot#: C3A4BR054), CYP2B6 (Lot#: C2B6BR057), and CYP2C8 (Lot#: C2C8BR008A), were acquired from Cypex for enzymatic activity studies. 48-well (Lot#: 11520012) and 96-well (Lot#: 31219012) Collagen I-pre-coated plates were purchased from Corning.

### Plateable hepatocytes cytotoxicity

Cryopreserved plateable rat and human hepatocytes were thawed by immersing vials in a pre-warmed 37 °C water bath. The cell pellets were then transferred to thawing medium and centrifuged to isolate viable cells. Then, hepatocytes were diluted to 0.1 million cells mL^-1^ and seeded into 96-well plates (0.1 mL *per* well) in triplicate. After 6 hours of attachment, the plating medium was replaced with incubation medium containing 1 % Matrigel™. On the following day, the cell medium was replaced with a series of metoprolol dosing solutions (0, 100, 150, 200, 250 and 500 μM) in the presence or absence of 50 μM 1-aminobenzotriazole (ABT), a cytochrome P450 inhibitor, and then the hepatocyte cytotoxicity was determined using a commercially available ATP detection assay (CellTiter-Glo®). Prior to the measurement, the cell culture plate was equilibrated to ambient temperature. Subsequently, 100 μL of CellTiter-Glo® reagent was dispensed into each well, followed by orbital shaking (5 min) to ensure complete cell lysis. After a 10-minute stabilization at room temperature, luminescence signals were quantified using an envision® multilabel plate reader.

### Plateable hepatocytes stability

Cryopreserved human hepatocytes were thawed by immersing vials in a pre-warmed 37 °C water bath. The cell pellets were then transferred to thawing medium and centrifuged to isolate viable cells. Then, hepatocytes were diluted to 0.7 million cells mL^-1^ and seeded into collagen-coated 48-well plates (0.2 mL *per* well) in triplicate. After 6 hours of attachment, the plating medium was replaced with incubation medium containing 1 % Matrigel™. On the following day, the medium was replaced with a dosing solution containing 1 μM metoprolol to initiate metabolic reactions. Aliquots were collected at 0, 2, 4, 8, 24, 48 and 72 hours, and reactions were quenched with cold acetonitrile/methanol (1:1, v/v) containing labetalol (100 ng mL^-1^). After centrifugation (12,000*g*, 15 min, 4 °C), supernatants were analysed *via* LC-30AD-API5500 (LC-MS/MS, SCIEX, USA) for semiquantitative assessment.

### Recombinant cytochrome P450 reaction phenotyping

Human recombinant enzymes were diluted to 50 pmol mL^-1^ in 50 mM phosphate buffer. Metoprolol was added to the enzyme solution (final concentration: 1 μM) in duplicate and preincubated for 5 min. Reactions were initiated by adding NADPH (final concentration: 1 mM) and aliquots were collected at 0, 15, 30, 60 and 120 min. Samples were quenched with cold acetonitrile containing tolbutamide (10 ng/mL), centrifuged (12,000*g*, 15 min, 4 °C) and analysed by LC-MS/MS. For qualitative metabolite analysis, 0- and 60-minute samples were dried, reconstituted, centrifuged (12,000*g*, 15 min, 4 °C) and analysed on Ultimate 3000-Q-Exactive Plus (LC-HRMS, ThermoFisher, Germany).

### Metabolite identification in recombinant cytochrome 2D6 enzymes and plateable hepatocytes

Human recombinant cytochrome 2D6 enzymes were diluted to 50 pmol mL^-1^ in 50 mM phosphate buffer. Metoprolol was added to the enzyme solution (final concentration: 1 μM) in duplicate and preincubated for 5 min. Reactions were initiated by adding NADPH (final concentration: 1 mM), and aliquots were collected at 60 min. Samples were quenched with cold acetonitrile containing 0.1 % formic acid, centrifuged (12,000*g*, 15 min, 4 °C), then supernatants were dried, reconstituted, centrifuged (12,000*g*, 15 min, 4 °C), and subjected to an analysis on LC-HRMS..

Cryopreserved rat, dog, monkey and human hepatocytes were thawed by immersing vials in a pre-warmed 37 °C water bath. The cell pellets were then transferred to thawing medium and centrifuged to isolate viable cells. Then, hepatocytes were diluted to 0.7 million cells mL^-1^ and seeded into collagen-coated 48-well plates (0.2 mL *per* well) in duplicate. After 6 hours of attachment, the plating medium was replaced with incubation medium containing 1 % Matrigel™. On the following day, the medium was replaced with a dosing solution containing 10 μM metoprolol to initiate metabolic reactions. Reactions were quenched at 72 hours with cold acetonitrile containing 0.1 % formic acid, and supernatants were dried under nitrogen gas. The residues were reconstituted in acetonitrile/water (1:9 volume ratio) with 0.1 % formic acid and analysed LC-HRMS. The M7 metabolite was isolated, purified, and concentrated via a mass spectrometry switching valve, followed by sample reconstitution, and subsequent analysis on LC-HRMS.

### Semi-quantitative and qualitative analysis

Semi-quantitative LC-MS/MS analysis was performed using an LC30 pump (Shimadzu, Japan) coupled to an API 5500 mass spectrometer. The mobile phase A was distilled water containing 0.1 % formic acid and the mobile phase B was acetonitrile containing 0.1 % formic acid. The LC gradient was as follows: 0 to 0.80 min at 5 % B; 0.80 to 1.20 min, 5 to 95 % B; 1.20 to 1.80 min at 95 % B; 1.80 to 1.81 min, 95 to 5 % B; and 1.81 to 2.5 min at 5 % B (flow rate: 0.8 mL min^-1^). MS parameters included: curtain gas: 2.76 MPa, gas 1: 2.76 MPa, gas 2: 4.14 MPa, ion source voltage: 5500 V, source temperature: 550 °C, auxiliary nitrogen pressure: 0.7 MPa, and entrance potential voltage: 10 eV. Data was acquired in multiple ions monitor mode using Analyst 1.6.3 software (SCIEX, USA; https://sciex.com/products/software/analyst-software).

Qualitative LC-HRMS analysis was conducted using an Ultimate 3000 pump (ThermoFisher, Germany) and a Q-Exactive Plus spectrometer. The mobile phase A was distilled water containing 0.1 % formic acid and the mobile phase B was acetonitrile containing 0.1 % formic acid. The liquid chromatography (LC) gradient spanned 22 min: 0 to 1.0 minute at 5 % B; 1.0 to 6.0 min, 5 to 15 % B; 6.0 to 14.0 mi, 15 to 25 % B; 14.0 to 16.0 min, 25 to 90 % B; 16.0 to 18.0 min, 90 to 95 % B; 18.0 to 20.0 min, 95 to 5 % B; and 20.0 to 22.0 min at 5 % B (flow rate: 0.3 mL min^-1^). MS parameters included: spray voltage: 3.8 kV, capillary temperature: 320 °C, heater temperatures: 350 °C, sheath gas: 2.76 MPa, auxiliary nitrogen pressures: 0.7 MPa and tube lens voltage: 10 eV. The full scan Q1 resolution was set to 70,000, and the automatic gain control (AGC) target value was set to 5×10^5^ intensity. The maximum injection time was limited to 50 ms. The dd-MS^2^ resolution was set at 17,500, with the AGC target set at 5×10^4^. External calibration was performed, yielding a deviation of less than 5.0 *ppm*. Data was processed using Xcalibur 4.1.31.9 software (ThermoFisher, Germany; https://www.thermofisher.com/order/catalog/product/OPTON-30965).

### Data analysis

LC-MS/MS raw data was analysed using Analyst software, and hepatic clearance results (mean±SD) was processsed with GraphPad Prism 5.0 (GraphPad software, USA; https://www.graphpad.com/). Phenotyping data (mean values) was analysed using Microsoft Excel 2021 (mean values). Hepatic clearance was determined by quantifying metoprolol depletion following incubation with hepatocytes. The *in vivo* hepatic clearance was subsequently extrapolated using the well-stirred model incorporating intrinsic clearance [70-72]. Hepatocyte viability data was expressed as mean±SD. The statistical significance was determined by Student's *t*-test using GraphPad Prism. LC-HRMS raw data was extracted with Xcalibur, and metabolites were identified using Compound Discoverer 3.0 (ThermoFisher, Germany; https://www.thermofisher.com/order/catalog/product/OPTON-31061) by comparing fragment patterns between parent compounds, metabolites, and standards.

## Results and discussion

### Hepatocytes viability, metabolic stability and phenotyping study

**Figure 1.**
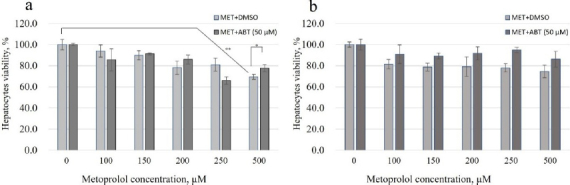
(a) rat and (b) human hepatocytes viability after 48 hours of treatment with metoprolol with or without the presence of ABT. *p < 0.05, **p < 0.01

The cytotoxic effects of metoprolol were assessed in plateable rat and human hepatocytes using the CellTiter-Glo® Luminescent Cell Viability Assay. In rat hepatocytes, metoprolol exhibited weak cytotoxicity, with significant reductions (>30 %) in cell viability observed at 500 μM (Figure 1a). 1-Aminobenzotriazole was a classic inhibitor of CYP450 enzymes; pre-treatment with ABT significantly increased the viability of rat hepatocytes and reversed the cytotoxic effect at 500 μM. In contrast, human hepatocytes demonstrated greater resistance to metoprolol-induced cytotoxicity (Figure 1b). No significant reduction in cell viability was observed across the tested concentration range (0 to 500 μM), regardless of ABT co-treatment.

**Figure 2.**
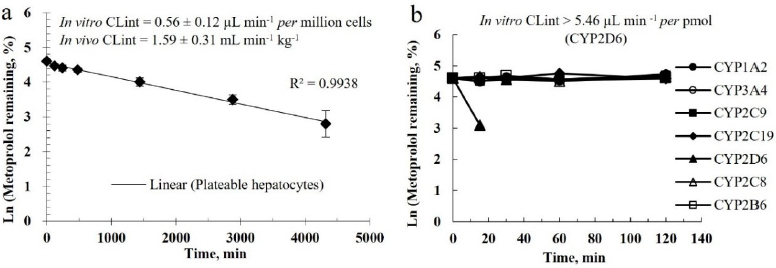
Metoprolol Remaining *vs.* incubation time in (a) plateable hepatocytes and (b)recombinant enzymes

These metabolic stability studies demonstrated that metoprolol exhibited typical low-clearance characteristics in plateable hepatocytes, with an *in vitro* intrinsic clearance (CL_int_) of 0.56±0.12 μL min^-1^ *per* million cells (Figure 2a) and a corresponding *in vivo* CL_int_ of 1.59±0.31 mL min^-1^ kg^-1^. These values are significantly below the 10 mL min^-1^ kg^-1^ threshold for low-clearance compounds and align well with previously reported [20,54] with a low clearance of 2.2 ± 0.7 μL min^-1^ *per* million cells in plateable human hepatocytes, correlating with an *in vivo* clearance of 1.4 mL min^-1^ kg^-1^ [20] (or 4.28 μL min^-1^ *per* million cells [73]), making the plateable hepatocytes particularly valuable for evaluating low-clearance compounds [74].

Enzyme phenotyping studies demonstrated remarkable specificity in metoprolol metabolism. As illustrated in Figure 2b, among the cytochrome P450 isoforms tested (CYP1A2, 2C9, 2C19, 3A4, 2B6 and 2C8), only CYP2D6 displayed significant metabolic activity, contributing to 100 % of the observed clearance with an intrinsic clearance rate of 5.46 μL min^-1^ *per* pmol protein. This finding corroborates prior reports on the exclusive involvement of CYP2D6 in metoprolol metabolism [58], highlighting its critical clinical implications. Given this highly selective CYP2D6-dependent metabolic pathway, these results emphasize the importance of genotype-guided dosing strategies, particularly in populations with a high prevalence of CYP2D6 poor metabolizers.

### Chromatographic and mass spectrometric characteristics of metoprolol and its metabolites

**Figure 3.**
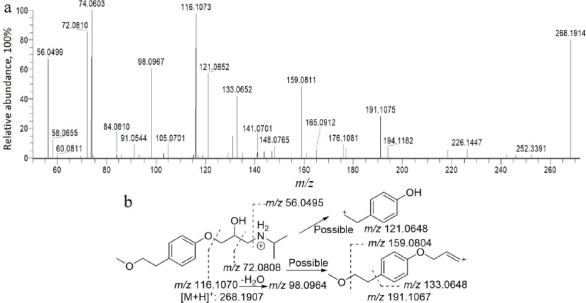
Collision-induced dissociation spectrum (**a**) and structural analysis (**b**) of metoprolol

In Figure 3, metoprolol was determined with a protonated molecular ion peak at *m*/*z* 268.1914. The collision-induced dissociation (CID) product ion spectrum exhibited characteristic fragment ions at *m*/*z* 56.0499, 72.0810, 98.0967, 116.1073, 121.0652, 133.0652, 159.0811 and 191.1075, supporting its structural identification. The LC analysis showed a retention time of approximately 10.86 min for the parent compound.

**Figure 4.**
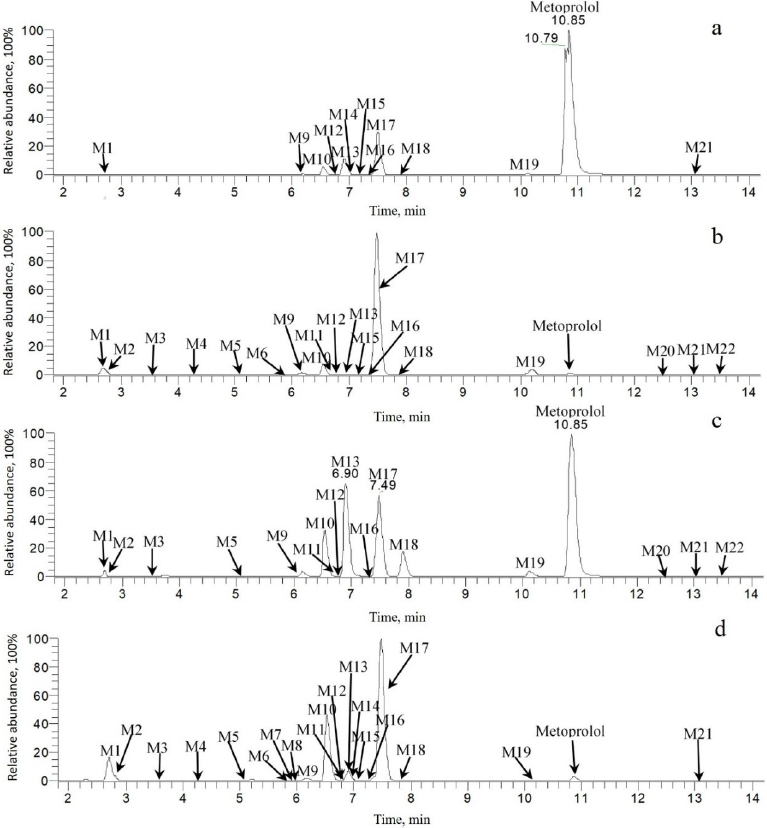
Selected ion chromatograms of metoprolol and its metabolites in plateable human (a), monkey (b), dog (c) and rat (d) hepatocytes

As summarized in Table 1 and illustrated in Figure 4, twenty-two metabolites were detected and fifteen were identified as new in plateable hepatocytes, eluting between 2.64 and 13.52 min, with effective chromatographic separation. Species-dependent metabolic profiles were observed in the metabolite identification results in plateable hepatocytes.

**Table 1.** Observed metabolites of metoprolol in recombinant 2D6 enzymes and plateable hepatocytes

ID	RT, min	*m*/*z* (+)		ME, *ppm*	Metabolic pathway	Relative peak area, %					Ref.
		Theoretical	Detecsted			Plateable hepatocytes				2D6^a^	
						R	D	Mk	H	H	
M1	2.65	270.1700	270.1711	4.1	*O*-demethylation and mono-oxygenation	8.8	0.8	4.7	D	26.6	-
M2	2.72	284.1492	284.1503	3.9	Oxidation and mono-oxygenation	0.9	D	0.1	ND	ND	-
M3	3.65	212.1281	212.1288	3.3	*O*-demethylation and *N*-dealkylation	D	0.7	0.1	ND	1.6	-
M4	4.44	226.1074	226.1082	3.5	*N*-dealkylation and oxidation	D	ND	D	ND	ND	-
M5	5.08	268.1543	268.1552	3.4	*O*-demethylation, mono-oxygenation, and dehydrogenation	0.6	D	0.1	ND	2.0	-
M6	5.82	444.1864	444.1878	3.2	*O*-demethylation, mono-oxygenation, dehydrogenation and glucuronidation	0.1	ND	0.1	ND	ND	-
M7	5.86	589.2538	589.2511	-4.6	Mono-oxygenation and glutathionylation (displacement)	D	ND	ND	ND	ND	[63]
M8	5.96	270.1700	270.1713	4.8	*O*-demethylation and mono-oxygenation	0.1	ND	ND	ND	ND	-
M9	6.12	254.1387	254.1395	3.1	Oxidation	0.4	D	0.2	D	1.7	[69]
M10	6.42	284.1856	284.1863	2.5	Mono-oxygenation	24.4	9.4	5.6	2.7	20.1	[62,63, 66-69,73]
M11	6.57	430.2072	430.2085	3.0	*O*-demethylation and glucuronidation	0.1	D	0.2	ND	ND	-
M12	6.70	430.2072	430.2082	2.3	*O*-demethylation and glucuronidation	D	D	0.2	D	ND	-
M13	6.79	254.1751	254.1755	1.6	*O*-demethylation	3.8	20.5	0.1	6.0	22.8	[62,63, 66,69,73]
M14	6.97	589.2538	589.2511	-4.6	Mono-oxygenation and glutathionylation (displacement)	D	ND	ND	D	ND	[63]
M15	7.19	444.1864	444.1875	2.5	*N*-dealkylation, acetylation and glucuronidation	D	ND	0.5	0.2	ND	-
M16	7.27	282.1700	282.1709	3.2	Mono-oxygenation and dehydrogenation	1.3	0.1	D	D	0.5	-
M17	7.36	268.1543	268.1551	3.0	Oxidation	57.2	19.9	82.6	14.6	18.3	[57]
M18	7.80	226.1438	226.1443	2.2	*N*-dealkylation	0.1	5.8	D	D	6.4	[69]
M19	10.16	444.2228	444.2236	1.8	Glucuronidation	D	D	4.2	0.7	ND	-
P	10.66	268.1907	268.1914	2.6		2.1	42.5	1.2	75.8	ND	-
M20	12.52	460.2177	460.2189	2.6	Mono-oxygenation and glucuronidation	ND	0.1	D	ND	ND	-
M21	13.05	258.1336	258.1340	1.5	*N*-dealkylation and di-oxygenation	0.1	0.2	D	D	ND	-
M22	13.52	460.2177	460.2189	2.6	Mono-oxygenation and glucuronidation	ND	D	0.1	ND	ND	-

ID - identity; RT – retention time; ME - mass error; 2D6^a^ - recombinant 2D6 enzymes; D - detected in trace amount; ND - not detected; R -rat; D -dog; Mk - monkey; H - human. The relative peak area abundance of the parent and metabolite was calculated based on their selected ion chromatographic peak areas.

**Figure 5.**
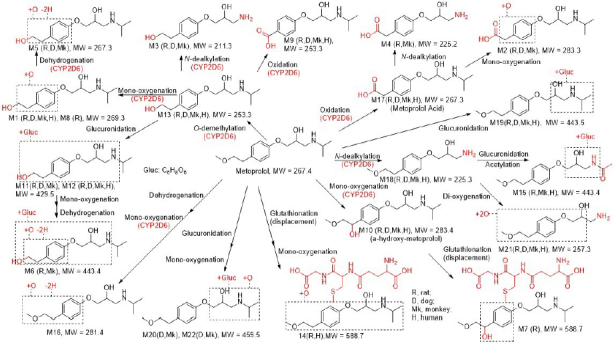
Proposed metabolic pathways of metoprolol in plated hepatocytes and CYP2D6 recombinant enzymes

In rat hepatocytes, M10 and M17 predominated, constituting 24.4 and 57.2 % of the total mass abundance, respectively. In dog hepatocytes, M13 and M17 were the major metabolites, accounting for 20.5 and 19.9 %. Notably, in monkey and human hepatocytes, M17 was the dominant metabolite, representing 82.6 and 14.6 % of the total mass abundance, respectively. According to the metabolite identification result in recombinant CYP2D6 enzymes, the most abundant metabolites were M1, M10, M13, and M17, with relative abundances of 26.6, 20.1, 22.8 and 18.3 %, respectively. Across all tested species and matrices, the major metabolic pathways consisted of mono-oxygenation, *O*-demethylation, and oxidation, with CYP2D6 confirmed as the principal enzyme mediating these biotransformations as shown in Figure 5.

### Metabolites elucidation

Metabolite structures were deduced based on mass defect analysis and fragmentation patterns.

#### Oxygenation derivatives

M10 exhibited a protonated molecular ion at *m*/*z* 284.1863; its characteristic ions were observed at *m*/*z* 56.0500, 74.0603, 98.0968, 116.1074, and specifically at *m*/*z* 207.1025, indicating that M10 was a mono-oxygenation metabolite. M10 eluted at a retention time of 6.55 min, which was close to that of α-hydroxy-metoprolol. Furthermore, M10 and α-hydroxy-metoprolol shared similar fragment ions at *m*/*z* 74.0600, 98.0964, 116.1069, 133.0647 and 207.1015, suggesting they were closely related.

M16 exhibited a protonated molecular ion at *m*/*z* 282.1709, with characteristic ions at *m*/*z* 173.0601, 205.0869 and 240.1228, indicating that M16 was a metabolite of mono-oxygenation and dehydrogenation.

#### O-demethylation products

M13 exhibited a protonated molecular ion at *m*/*z* 254.1755, with characteristic ions at *m*/*z* 116.1072, 159.0808 and 177.0914, indicating that M13 was an *O*-demethylation metabolite.

M1 and M8 displayed the same protonated molecular ion at *m*/*z* 270.171, along with identical characteristic ions at *m*/*z* 175.0760 and 193.0866, suggesting a mono-oxygenation reaction on M13.

M3 presented a protonated molecular ion at *m*/*z* 212.1288, with characteristic ions at *m*/*z* 74.0603, 159.0810, and 177.0915. No modification was detected on the *m*/*z* 177.0915 moiety, and *N*-dealkylation was confirmed by the indirect fragment at *m*/*z* 74.0603, which was dealkylated from *m*/*z* 116.1072, therefore, M3 was classified as a metabolite of both *O*-demethylation and *N*-dealkylation.

M5 displayed a protonated molecular ion at *m*/*z* 268.1552, with characteristic ions at *m*/*z* 116.1074, 191.0708, and 226.1079. No modification was detected on the *m*/*z* 116.1074 moiety, while dehydrogenation was observed on the moieties at *m*/*z* 191.0708 and *m*/*z* 226.1079 compared to M1 or M8, therefore, M5 was identified as a dehydrogenation metabolite from M1 or M8.

#### Oxidative transformations

M9 exhibited a protonated molecular ion at *m*/*z* 254.1395, its characteristic ions were observed at *m*/*z* 116.1075, 151.0396, 133.0653, and 177.0553, indicating that M9 was an oxidation metabolite.

M17 exhibited a protonated molecular ion at *m*/*z* 268.1551, its characteristic ions were observed at *m*/*z* 116.1073, 145.0653, 191.0708, and 226.1082. A special transition of loss of carboxyl group from *m*/*z* 191.0708 to *m*/*z* 145.0653 suggested that M17 was an oxidation metabolite. Furthermore, M17 was eluted at a retention time of 7.52 min, which was close to that of metoprolol acid, and they shared similar fragments at *m*/*z* 56.0498, 98.0964, 116.1069, 145.0647, 191.0701, 226.1073, and 250.1437. Therefore, M17 was identified as metoprolol acid.

M2 displayed a protonated molecular ion at *m*/*z* 284.1503, with characteristic ions at *m*/*z* 116.1074, 189.0552, and 207.0659. No modifications occurred on the *m*/*z* 116.1074 moiety, suggesting that M2 was a mono-oxygenation metabolite originating from M17.

M4 exhibited a protonated molecular ion at *m*/*z* 226.1082, with characteristic ions at *m*/*z* 56.0499, 74.0603, 145.0652, and 191.0709. No modifications were observed on the *m*/*z* 191.0709 moiety, indicating that M4 was an *N*-dealkylation metabolite derived from M17.

#### *N*-dealkylation modifications

M18 exhibited a protonated molecular ion at *m*/*z* 226.1443, with characteristic ions at *m*/*z* 74.0603 and *m*/*z* 191.1072. No modifications were detected on the *m*/*z* 191.1072 moiety, and *N*-dealkylation was confirmed by a transition from *m*/*z* 116.1072 to *m*/*z* 74.0603. Therefore, M18 was identified as a metabolite resulting from *O*-demethylation and *N*-dealkylation.

M21 presented a protonated molecular ion at *m*/*z* 258.1340, with a characteristic ion at *m*/*z* 209.0814, indicating that M21 was a di-oxygenation metabolite of M18.

##### Glucuronide conjugates

M6 showed a protonated molecular ion at *m*/*z* 444.1878, with characteristic ions at *m*/*z* 116.1073, 226.1074, and 268.1547, indicating that M6 was a metabolite of *O*-demethylation, mono-oxygenation, dehydrogenation and glucuronidation.

M11 and M12 exhibited the same protonated molecular ion at *m*/*z* 430.208, along with a common characteristic ion at *m*/*z* 254.176, which showed a significant transition of glucuronidation loss; thus, they were identified as glucuronidation metabolites derived from M13.

M15 displayed a protonated molecular ion at *m*/*z* 444.1875, with characteristic ions at *m*/*z* 191.0709 and 268.1550. No modifications were observed on the *m*/*z* 191.1075 moiety, and a glucuronide loss (-176 amu) was detected from *m*/*z* 444.1875 to 268.1550. Consequently, M15 was characterized as a metabolite of acetylation and glucuronidation derived from M18.

M19 displayed a protonated molecular ion at *m*/*z* 444.224, with characteristic ions at *m*/*z* 116.1073 and 268.1914. A notable glucuronide loss (-176 amu) was detected, transitioning from *m*/*z* 444.224 to 268.191, leading to its identification as a glucuronidation metabolite.

M20 and M22 displayed the same protonated molecular ion at *m*/*z* 460.219, and they exhibited identical characteristic ions at *m*/*z* 114.092 and 284.186, indicating that they were metabolites resulting from mono-oxygenation and glucuronidation.

##### Glutathione conjugates

**Figure 6.**
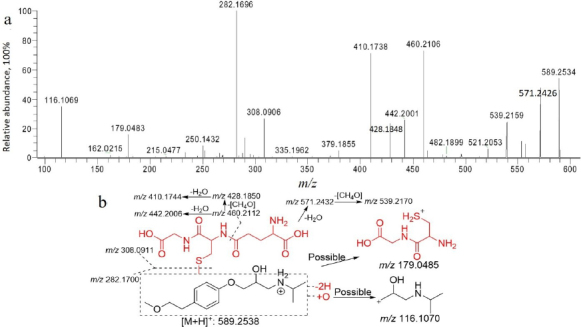
Collision-induced dissociation spectrum (a) and structural analysis (b) of M7

M7 and M14 exhibited a common protonated molecular ion at *m*/*z* 295.1287 (*z* = 2). The characteristic ions observed were *m*/*z* 130.0498, 179.0483, 308.0923 and 282.1696. Considering these ions at *m*/*z* 130.0498, 179.0483 and 308.0923 were characteristic fragments of glutathione conjugates. Moreover, mass spectrometric analysis of the concentrated M7 metabolite showed enhanced glutathione-associated fragment peaks (Figure 6), providing evidence for potential glutathione adduct formation. Therefore, M7 and M14 were metabolites resulting from mono-oxygenation and glutathionylation (displacement).

Despite its classification as a low clearance drug, the comprehensive metabolic fate of metoprolol remains incompletely characterized in current literature. Notably, our study has identified up to 22 metabolites in plateable hepatocytes, as shown in Table 1, significantly expanding upon previously identified metabolites [62-69,75,76]. Tao *et al* [62] identified two metabolites, α-hydroxy-metoprolol (*m*/*z* 116.1, 133.1, 163.1 and 207.1) and *O*-desmethyl-metoprolol (*m*/*z* 98.1, 116.1, and 177.1). Both metabolites were also detected in plateable hepatocytes, with more abundant fragments for α-hydroxy-metoprolol (*m*/*z* 56.0500, 74.0603, 86.0966, 98.0968, 116.1074, 133.0653, 163.0758, 207.1025 and 224.1289) and *O*-desmethyl-metoprolol (*m*/*z* 56.0499, 72.0810, 74.0602, 98.0966, 116.1072, 133.0651, 151.0757, 159.0808, 177.0914 and 212.1285). Li *et al.* [63] and Bae *et al.* [67] reported the formation of reactive glutathione adducts through the artificial addition of the xenobiotic glutathione cofactor. Consistent with this, two glutathione adducts were directly captured in plateable rat and human hepatocytes, demonstrating prolonged metabolic enzyme activity.

Aligning with the findings reported by Ma *et al.* [77], plateable hepatocytes effectively captured the low-clearance characteristics and pronounced interspecies differences in metoprolol metabolism after prolonged incubation. Human plateable hepatocytes maintained functional cytochrome P450 enzymes and accurately recapitulated metabolism mediated by human recombinant CYP2D6 enzymes, with principal metabolites exhibiting comparable relative abundance profiles (M10, M13, and M17). Notably, M10 was a minor metabolite in human plateable hepatocytes but constituted the major metabolite in rat plateable hepatocytes. Moreover, M10 demonstrated a tendency for further metabolism into a glutathione (GSH) conjugate in rat hepatocytes, suggesting potential toxicity associated with reactive metabolite formation. The involvement of CYP-mediated bioactivation in this toxic pathway was further supported by irreversible inhibition using 1-aminobenzotriazole, a non-specific CYP inhibitor, which substantiated the role of CYP2D6 in the observed hepatotoxic effects.

## Conclusion

In this study, metoprolol was characterized as a low-clearance compound based on its metabolic profile in plateable human hepatocytes. The plateable hepatocyte system demonstrated extensive metabolic capacity, with 22 metabolites of metoprolol identified for the first time. Functionally competent cytochrome P450 enzymes were maintained in human plateable hepatocytes, accurately recapitulating metabolite profiles generated by human recombinant CYP2D6 enzymes. The principal biotransformation pathways consisted of *O*-demethylation, mono-oxygenation and oxidative metabolism. Toxicologically, M10 was found to be metabolically converted into a glutathione (GSH) conjugate in rat plateable hepatocytes. The CYP-dependent nature of this hepatotoxic effect was confirmed through reversible inhibition using the non-specific CYP inhibitor 1-aminobenzotriazole. While weak hepatotoxicity occurred in rat hepatocytes at a supratherapeutic concentration of 500 μM, no significant hepatotoxicity was observed in human hepatocytes at metoprolol concentrations below 500 μM. Collectively, these findings validate plateable hepatocytes as a robust *in vitro* system for studying the metabolism of low-clearance compounds, offering valuable insights for preclinical drug safety assessment.

## Supplementary material

Additional data are available at https://pub.iapchem.org/ojs/index.php/admet/article/view/2961, or from the corresponding author on request.


