# Supplementary material for: Elucidating CYP2D6-driven metabolism and hepatotoxic bioactivation of metoprolol in plateable human and animal hepatocytes
Source: ADMET DMPK. 2025 Oct 30;13(5):2961. doi: 10.5599/admet.2961 (PMC12662248; doi:10.5599/admet.2961)
Supplement: Supplementary file 1 [file ADMET-13-2961-S1.pdf]

ADMET & DMPK 13(5) (2025) S2961

Open Access : ISSN : 1848-7718

http://www.pub.iapchem.org/ojs/index.php/admet/index

Supplementary material to

Elucidating CYP2D6-driven metabolism and hepatotoxic bioactivation of metoprolol in plateable human and animal hepatocytes

Jiang Pu, Mei Yang, Min Zhang, Ruiqi Gao, Yue Xiao, Lingyu Liu, Chuanjing Zhang, Wennuo Xu, Kaifang Li and Wanyong Feng

Bioduro Biologics Co., Ltd., Shanghai, China

ADMET & DMPK **13(5)** (2025) 2961; https://doi.org/10.5599/admet.2961

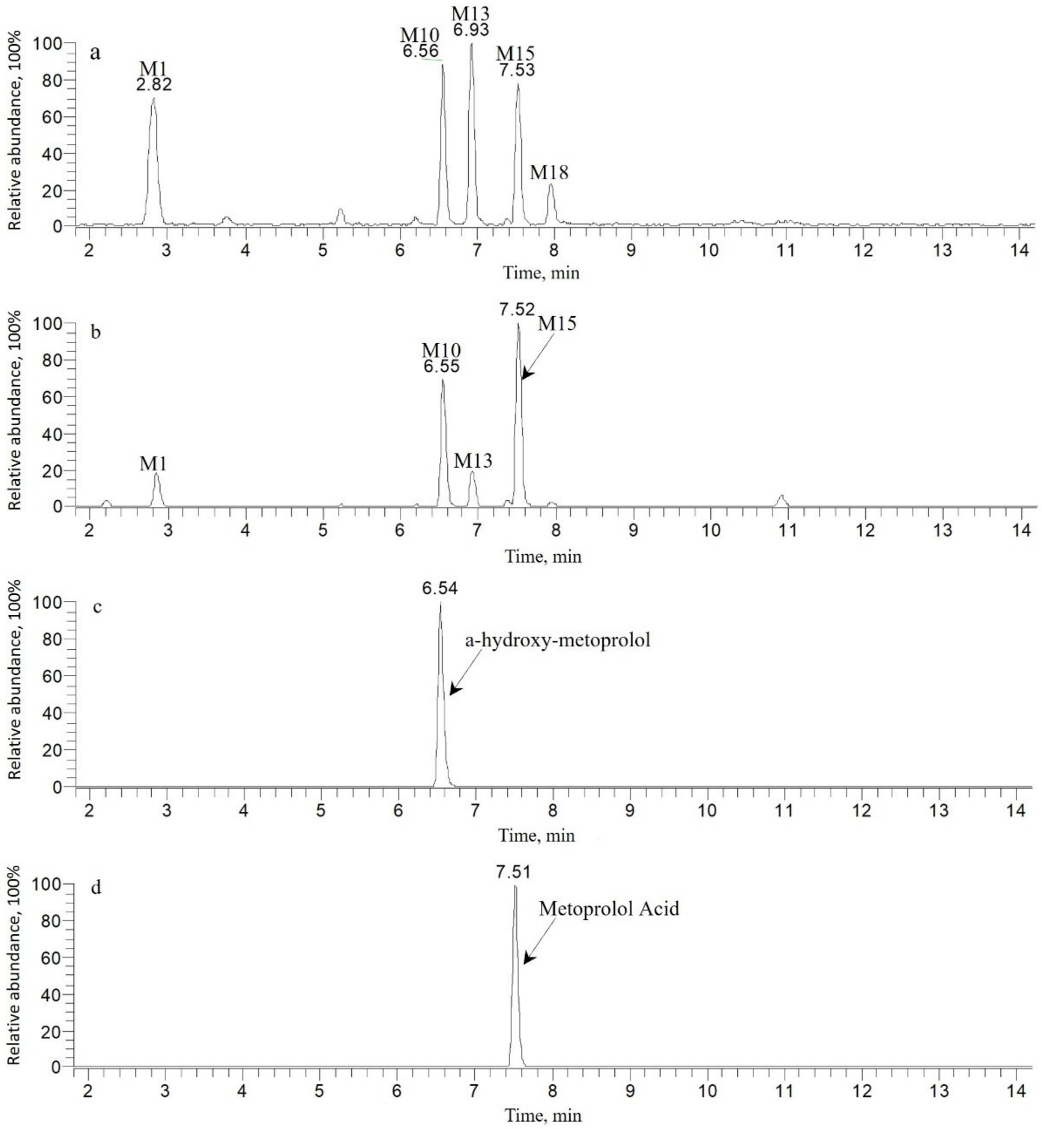

Figure S1. Selected ion chromatograms of metoprolol and its metabolites in recombinant enzymes (a) and rat hepatocytes (b), a-hydroxy-metoprolol (c), and metoprolol acid (d)

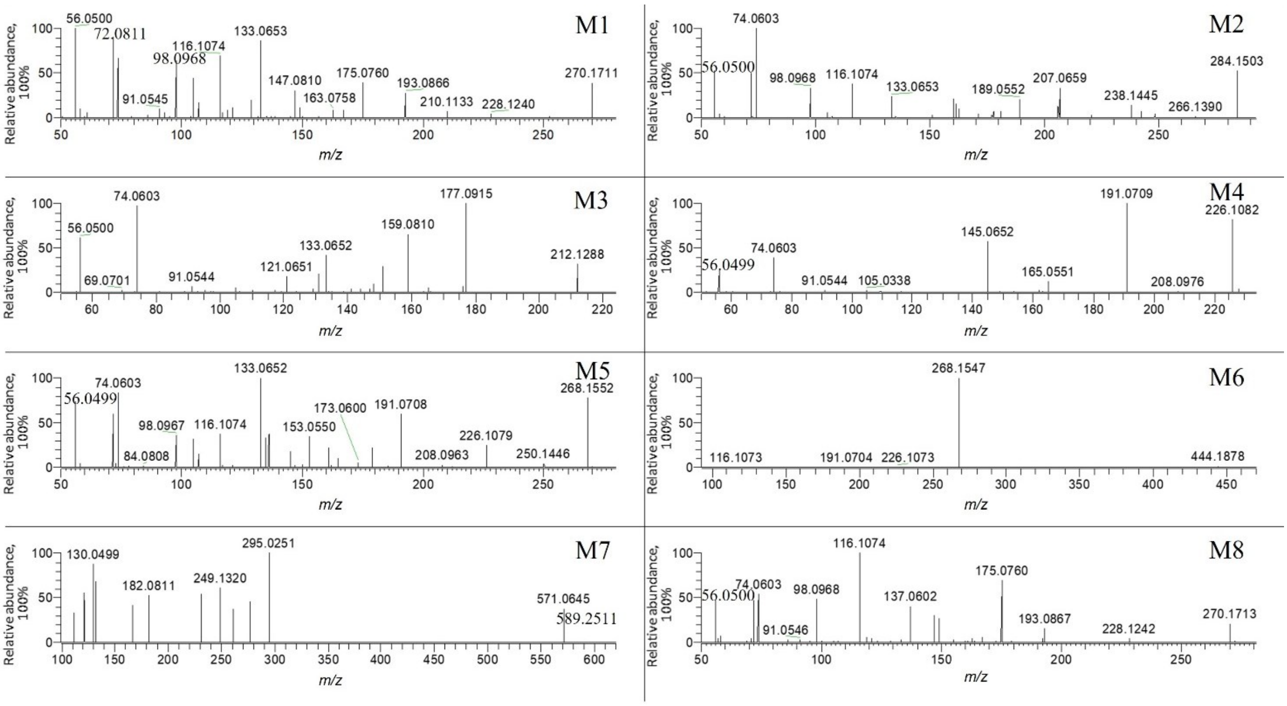

Figure S2. MS/MS spectra of metoprolol metabolites (M1 to M8)

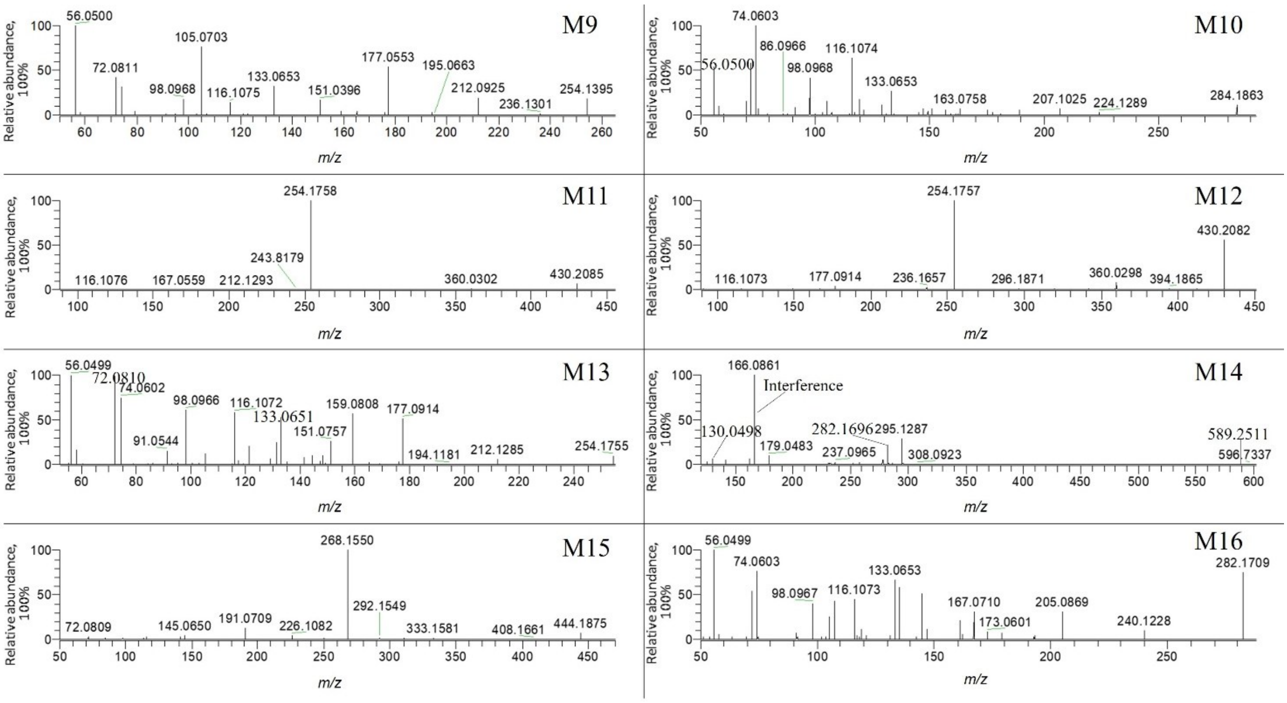

Figure S3. MS/MS spectra of metoprolol metabolites (M9 to M16)

CC BY

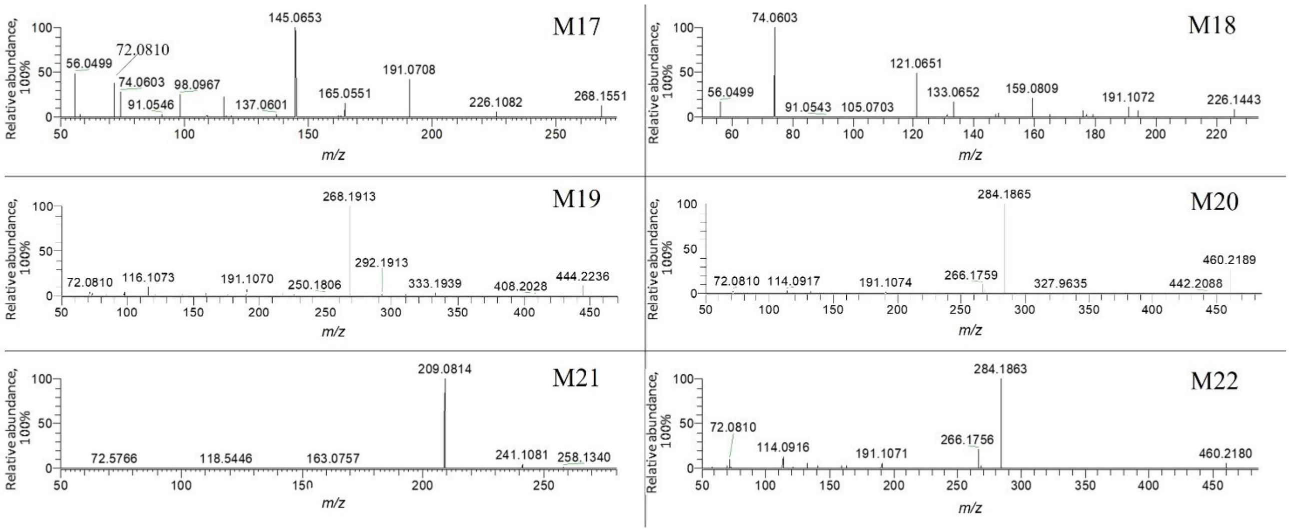

Figure S4. MS/MS spectra of metoprolol metabolites (M17 to M22)

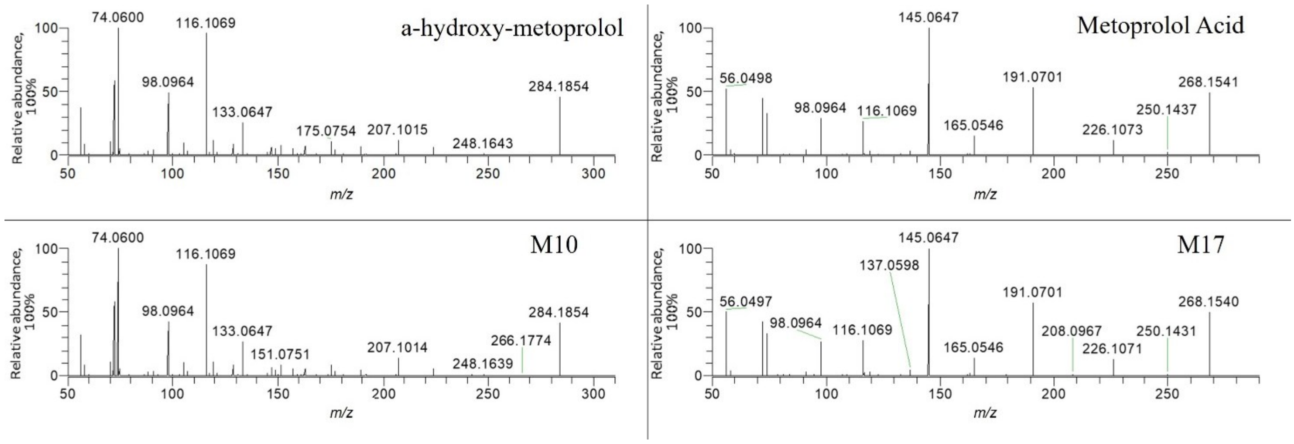

Figure S5. MS/MS spectra of a-hydroxy-metoprolol, metoprolol acid and metoprolol metabolites (M10 and M17)

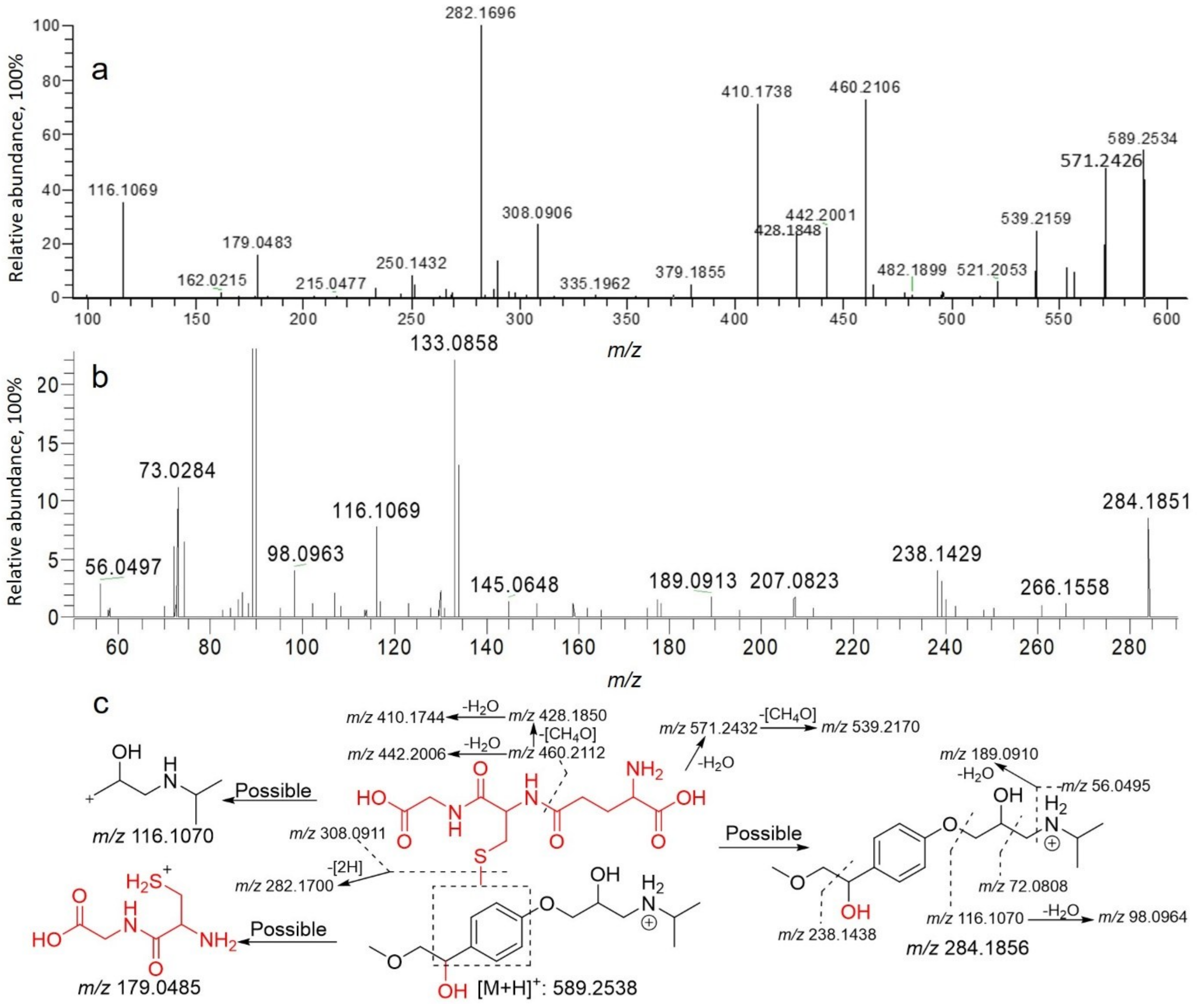

Figure S6. MS/MS spectra of M10 (a), In-source collisional *m/z* 284.1851 fragment of M10 (b) and structural analysis of M10 (c)

CC BY
